# Effect of *Pheretima aspergillum* on reducing fibrosis: A systematic review and meta-analysis

**DOI:** 10.3389/fphar.2022.1039553

**Published:** 2022-12-23

**Authors:** Tianren Xu, Xiaonan Liu, Shengguang Wang, Hongwei Kong, Xiaojun Yu, Congying Liu, Huaying Song, Peng Gao, Xin Zhang

**Affiliations:** ^1^ College of Pharmacy, Shandong University of Traditional Chinese Medicine, Jinan, China; ^2^ Institute of Pharmacy, Shandong University of Traditional Chinese Medicine, Jinan, China; ^3^ College of Traditional Chinese Medicine, Shandong University of Traditional Chinese Medicine, Jinan, China

**Keywords:** *Pheretima aspergillum*, anti-fibrosis, meta-analysis, preclinical evidence, animal studies

## Abstract

**Background:** *Pheretima aspergillum* (common name: Earthworm, Chinese name: dilong) has been used in traditional Chinese medicine for thousands of years. Recently, a few scientific studies have investigated the antifibrotic effects of Dilong extract (DE) and produced controversial results. We conducted a meta-analysis to make an informed decision on the antifibrotic effects of Dilong extract.

**Methods:** The studies on antifibrotic effects of Dilong extract published until July 2022 in the scientific databases [PubMed, Web of Science, China National Knowledge Infrastructure (CNKI), VIP database for Chinese Technical Periodicals, SinoMed and WanFang database] were reviewed. The RevMan 5.4.1 software was used for standardized mean difference (SMD) analysis. Two researchers independently reviewed all the studies, and their quality was assessed using the Cochrane risk of bias tool.

**Results:** A total of 325 studies were found in the scientific databases; however, only 13 studies met the criteria for analysis. Dilong extract treatment was associated with antifibrotic effects *via* inhibiting the transforming growth factor beta 1 (TGF-β1, SMD = −3.16, 95% CI: −4.18, −2.14, *p* < .00001) and alpha-smooth muscle actin (α-SMA: SMD = −2.57, 95% CI: −3.47, −1.66, *p* < .00001).

**Conclusion:** Dilong extract effectively reduces tissue fibrosis; thus, further scientific studies should be conducted to investigate and develop it for clinical use.

**Systematic Review Registration:** https://www.crd.york.ac.uk/prospero/, identifier CRD42022357141.

## 1 Introduction

Fibrosis is a process of progressive scar formation caused by tissue damage or inflammation, which can lead to organ damage and failure (Rockey et al., 2015). As a chronic disease, many pathogenic factors can cause, and tissue fibrosis is also the main cause of death and disability in many diseases (Zeisberg and Kalluri, 2013). Tissue fibrosis may occur in all organs of the human body. After tissue injury, it will trigger a series of inflammatory reactions, stimulate the continuous proliferation of related cells, and deposit a large amount of extracellular matrix, leading to the loss of physiological functions of organs (Friedman et al., 2013). Fibrosis can occur in organs such as the liver, lung, kidney, and heart. The antifibrotic mechanisms involve inhibiting specific cell pathways and cytokines secretion. Still, there is no effective treatment available for fibrosis (Xing et al., 2021). Polypeptides are gaining attention in research because of their strong bioactivity, low toxicity, highly selective and effective (Zhu et al., 2017). At present, only a small number of peptide drugs have entered the clinic directly with anti-fibrosis as an indication. However, more and more experimental studies have shown that peptide drugs can slow down the process of fibrosis (Li et al., 2019; Wang et al., 2021; Isakova et al., 2022; Song et al., 2022b), which means that anti-fibrosis peptide drugs have great development prospects. In the field of traditional Chinese medicine, the effective components of animal medicine are mostly polypeptides, such as earthworms, centipedes, sea cucumbers, etc.

In China, earthworms (*Pheretima aspergillum*), also known as Dilong, are reported to possess antifibrotic, anticoagulant, antithrombotic, antibacterial, antitussive, and antiasthmatic effects (Liu et al., 2019). The main antifibrotic ingredient in Dilong is polypeptide (Mihara et al., 1983). A meta-analysis was conducted on reported antifibrotic activities in animal (rat and mouse) models. The activity data were extracted and analysed using the RevMan5.4 (Review Manager software, version 5.4, Copenhagen: The Nordic Cochrane Centre, The Cochrane Collaboration, 2014) and Stata 16.0 (Stata statistical software, version 16.0, Stata Corporation, College Station, Texas). This study aims to provide concrete scientific evidence for the antifibrotic effects of earthworms on the lung, liver, and kidney through a meta-analysis and systematic review. The scientific evidence from this study can be used to make informed decisions on earthworms’ use in the clinic and further research directions.

## 2 Materials and methods

For the search method for this study, we have registered it on PROSPERO with the registration Id CRD42022357141. In order to improve the quality of this meta-analysis report, this paper refers to the PRISMA list, a normative guide in meta-analysis, taking into account all aspects of the items given in the list, the relevant list of which can be found in Supplementary Material S2. AMSTAR 2 is a newly developed methodological quality assessment tool for systematic evaluation, with a good practicality and can critically affect the validity of the conclusions. We added AMSTAR 2 to further evaluate the methodological quality of this meta-analysis, and specific item information will be presented in Supplementary Material S3.

### 2.1 Search strategy

The electronic databases [Web of Science, PubMed, China National Knowledge Infrastructure (CNKI), VIP Database for Chinese Technical Periodicals (VIP), SinoMed and WanFang] were searched by two independent researchers. All the databases were searched from their respective inception to July 2022. The following terms were used for the search: “fibrosis” OR “anti-fibrosis” AND “Dilong” OR “Earthworm” OR “*P. aspergillum*” OR “*Lumbricus terrestris*” OR “Eisenia worm” OR “*Eisenia foetida*.” Study selection was restricted to the English and Chinese languages. A detailed search strategy is presented in Supplementary Material S1.

### 2.2 Study selection

The inclusion criteria used for the selection of the studies are 1) randomised controlled animal experiments, 2) successful tissue fibrosis experimental model, 3) the experimental group receives earthworm extract, 4) availability of information on the extraction method, 5) availability of information on the molecular weight of the product, 6) availability of treatment time, and 7) control group can be without any intervention or oral administration of distilled water or saline. The exclusion criteria to select the studies are 1) repeated studies; 2) case reports, 3) reviews, 4) meta-analysis, 5) non-availability of the full text; 6) no information on the quantification of outcome indicators, and 7) no information on quantifiable outcome indicators.

### 2.3 Data extraction

Two researchers independently read all the studies, extracted data, and cross-checked. In case of ambiguities in arriving at a decision, they are resolved through discussions or consultation with a third party. The studies were screened to find the relevant ones in the following sequence: 1) title, 2) abstract, and 3) full text. The corresponding authors were contacted to obtain the missing information if any. The “Digram Designe” software was used to extract the data from charts. The details of the first author, publication year, country, animal species, interventions, treatment duration, experimental model, and quantification of output indicators were summarised in table format.

### 2.4 Methodological quality and assessment of studies

Two investigators independently applied the SYRCLE’s risk of bias tool for animal studies to evaluate the risk of bias and were cross-checked. If any, Ambiguities were solved through discussions and a third party’s consultation. The risk of bias was divided into “low,” “high,” and “uncertain” categories.

### 2.5 Data analysis

The research outcome indicators are continuous variables, and standardized mean difference (SMD) and its 95% confidence interval are used as the effect variables. Meta-analysis was performed using Revman5.4.1 software. If the heterogeneity of the results is small (I^2^ < 50%), the fixed effect model is used for meta-analysis. If the heterogeneity of the results is large (I^2^ ≥ 50%), the random effect model is used for meta-analysis. When heterogeneity is large, subgroup analysis is used to evaluate the source of heterogeneity. Stata16.0 software was used to plot the funnel plot, and the publication bias was evaluated using the Berg and Egger tests.

## 3 Results

### 3.1 Selection of the studies

**FIGURE 1:**
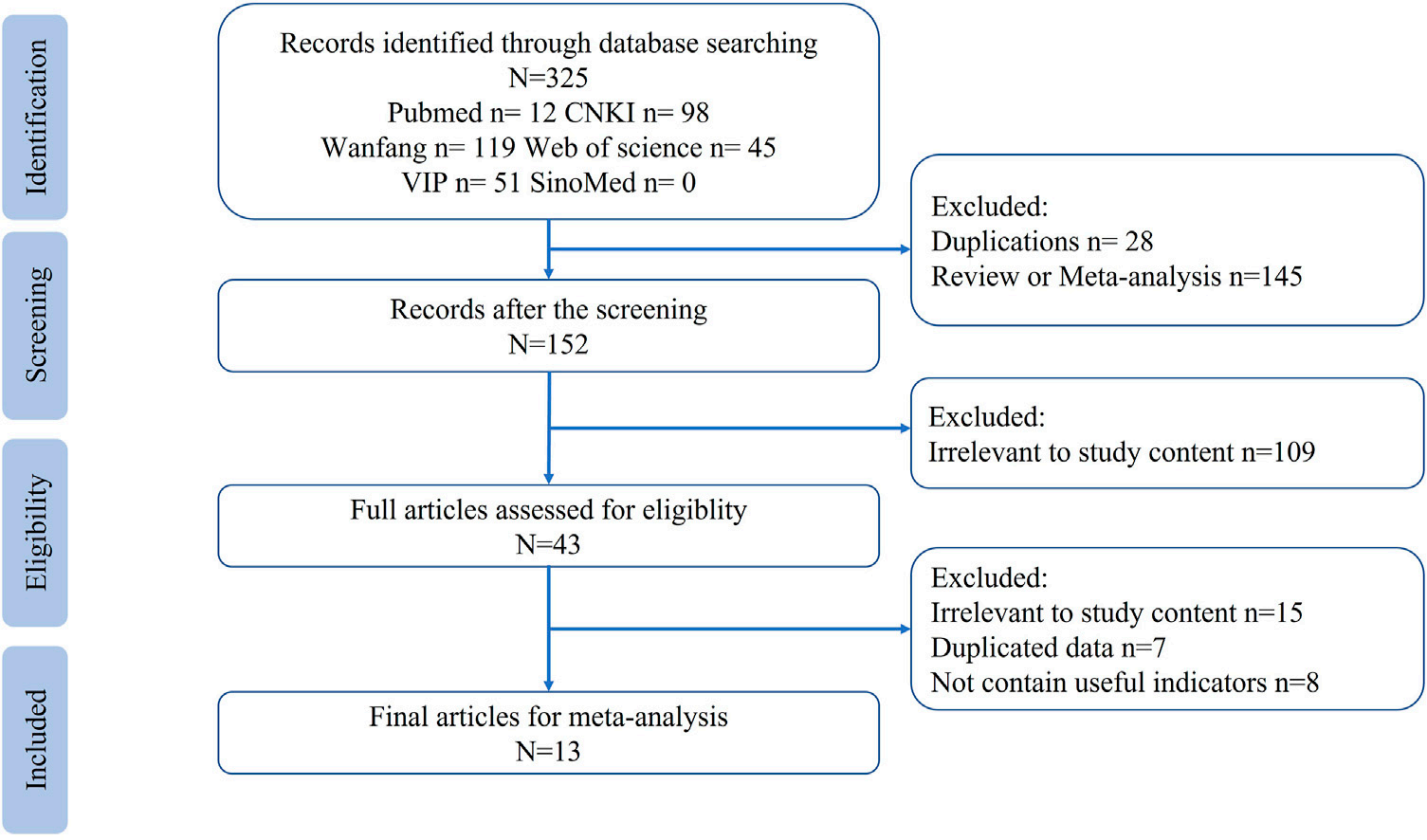
Flowchart of study search.

A total of 325 papers were obtained from the scientific databases, and only 13 studies met the inclusion and exclusion criteria (Chen et al., 2005a; Sheng et al., 2006; Luan, 2012; Tang, 2014; Zheng et al., 2015a; Yang et al., 2016; Ran, 2017; Zheng et al., 2017; Shen et al., 2018; Yu et al., 2018; Wang et al., 2019; Chen et al., 2020; Song et al., 2022a) and were included in the meta-analysis. The selection process and results are shown in Figure 1.

### 3.2 Characteristics of the included studies

The included studies used either sex of SD or Wistar rats and C57BL/6 or BALB/c mice. The studies reported the extraction methods and molecular weights of the components. The primary outcome biomarker was the expression of fibrosis-related proteins, including Transforming Growth Factor-β1 (TGF-β1) and α-Smooth Muscle Actin (α-SMA), and the secondary outcome was the inflammation score of fibrosis tissue. In the case of evaluating renal interstitial fibrosis, the Scr and BUN levels were also included. The characteristics of the included studies are shown in Table 1.

**TABLE 1:** Characteristics of studies used in meta-analysis.

Studies	Species	Age/Weight	Sample size	Intervention	Control	Outcome measurement	Sampling time	Country
Shen Shen 2018	SD rats	(180 ± 20)g	Ne = 30	lumbrokinase	Normal saline	The experission of TGF- β1 and α-SMA; the level of BUN and Scr	14 Days	China
			Nc = 20					
Li Sheng 2006	mice	18–22 g	Ne = 10	DE (water extract)	Normal saline	pulmonary index	28 Days	China
			Nc = 10					
Ting Yu 2018	SD Rats	(180 ± 20) g	Ne = 24	DE (water extract)	Normal saline	pulmonary index	28 Days	China
			Nc = 16					
Hong Chen 2005	Wister rats	(160 ± 10)g	Ne = 20	DE	Distilled water	The experission of TGF-β1 and α-SMA	8 weeks	China
			Nc = 16					
Minmin Zheng 2017	Wister Rats	180–200 g	Ne = 32	DE (Earthworm component serine proteolytic enzyme)	Distilled water	The experission of TGF-β1 and α-SMA	14 Days, 28 Days	China
			Nc = 16					
Minmin Zheng 2015	Wister Rats	180–200 g	Ne = 32	DE (Earthworm component serine proteolytic enzyme)	Distilled water	The level of BUN and Scr	14 Days, 28 Days	China
			Nc = 16					
Qiannan Song 2022	BALB/c mice	(210.75 ± 15.21)g	Ne = 10	DE (ethnaol extract)	Normal saline	The experission of TGF-β1 and α-SMA	5 weeks	China
			Nc = 10					
Hong Chen 2020	Mice	18–20 g	Ne = 10	DE	Distilled water	The experission of TGF- β1 and the degree of pulmonary fibrosis	14 Days	China
			Nc = 10					
Huihui Wang 2019	C57BL/6J mice, B	18–20 g	Ne = 30	DE (70% ethanol extract)	Normal saline	The expression of TGF-β1 and α- SMA	14 Days	China
			Nc = 10					
Siyan Ran 2017	mice	18–20 g	Ne = 15	DE	Normal saline	The level of BUN and Scr	12 weeks	China
			Nc = 15					
Qiufeng Tang 2014	Balb/c mice	18–20 g	Ne = 8	DE	Normal saline	Lung inflammation score	6 weeks	China
			Nc = 8					
Zhongqiu Luan 2012	Witar Rats	180–200 g	Ne = 16	DE (Earthworm component serine proteolytic enzyme)	Distilled water	The expression of TGF-β1 and α- SMA; the level of BUN and Scr	4 weeks	China
			Nc = 16					
Yang J. 2016	male C57BL/6 mice	20–25 g	Ne = 18	DE	Normal saline	Lung inflammation score	7 Days, 14 Days, 28 Days	China
			Nc = 18					

*DE, Dilong extracts; Ne, number of experiments; Nc, number of controls.

### 3.3 Quality evaluation of included studies

**FIGURE 2:**
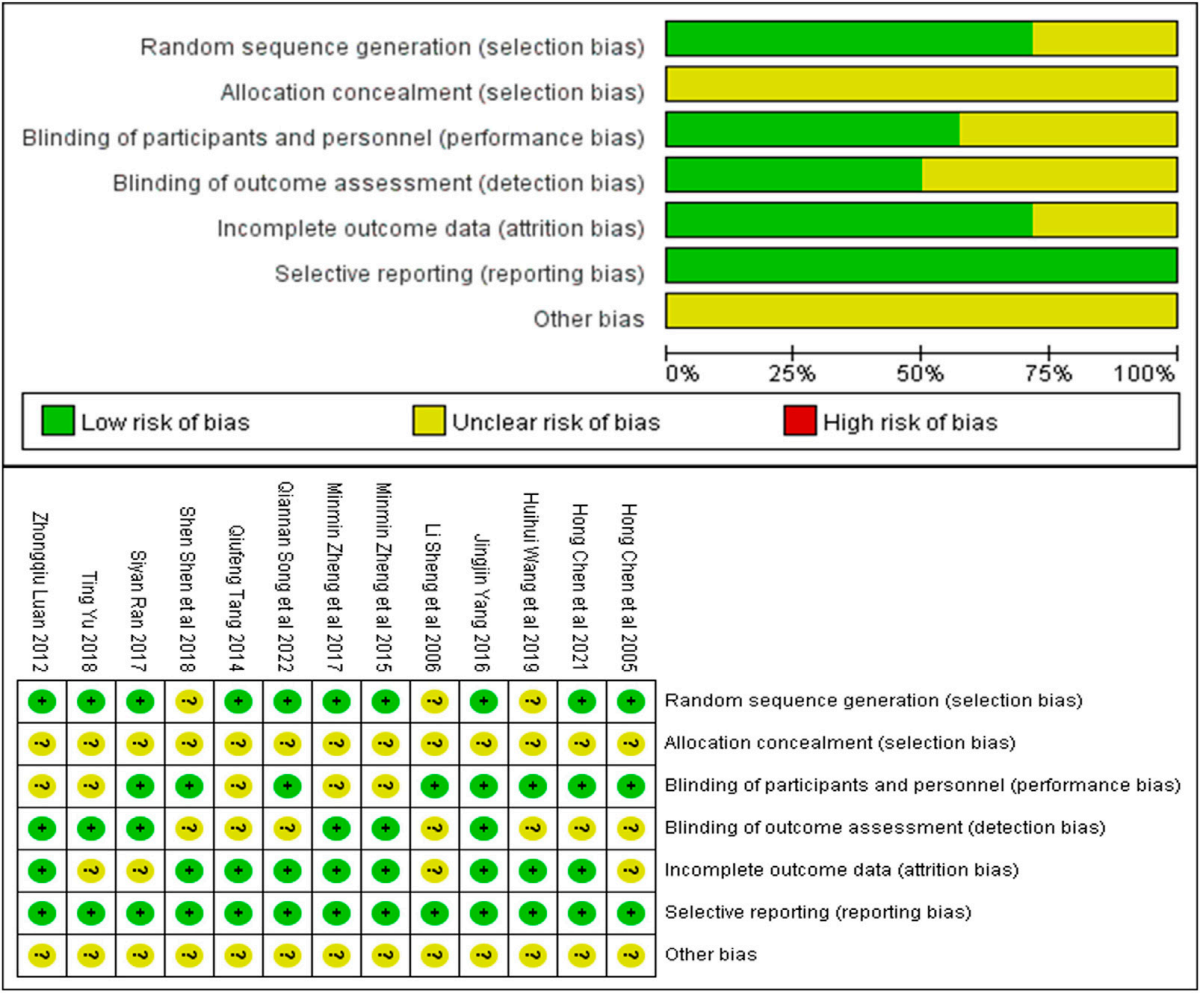
Risk of bias summary: Review authors’ judgments about each risk of bias item for each included study.

The quality of included studies was assessed using the SYRCLE animal experiment bias risk assessment scale and shown in Table 2. The overall quality of the included studies was medium. The Cochrane bias risk assessment tool was also used to evaluate the enrolled studies and the results are shown in Figure 2.

**TABLE 2:** SYRCLE’s tool for assessing the risk of bias in animal studies.

Studies	^a^Was the allocation sequence adequately generated and applied?	Were the groups similar at baseline or were they adjusted for confounders in the analysis?	^a^Was the allocation adequately concealed?	Were the animals randomly housed during the experiment?	Were the caregivers and/or investigators blinded from knowledge about intervention each animal received during the experiment?	Were animals selected at random for outcome assessment?	Was the outcome assessor-blinded?	^a^Were incomplete outcome data adequately addressed?	^a^Were reports of the study free of selective outcome reporting?	^a^Was the study apparently free of other problems that could result in a high risk of bias?
Ting Yu 2018	Low	Low	Unclear	Low	Unclear	Unclear	Low	Unclear	Low	Unclear
Qiannan Song 2022	Low	Low	Unclear	Low	Unclear	Unclear	Low	Low	Low	Unclear
Chen Hong 2021	Low	Low	Unclear	Low	Unclear	Unclear	Low	Low	Low	Unclear
Huihui Wang 2019	Unclear	Low	Unclear	Low	Unclear	Unclear	Low	Low	Low	Unclear
Shen Shen 2018	Unclear	Low	Unclear	Low	Unclear	Unclear	Low	Low	Low	Unclear
Siyan Ran 2017	Low	Low	Unclear	Low	Unclear	Unclear	Low	Unclear	Low	Unclear
Minmin Zheng 2017	Low	Low	Unclear	Low	Unclear	Unclear	Low	Low	Low	Unclear
Minmin Zheng 2015	Low	Low	Unclear	Low	Unclear	Unclear	Low	Low	Low	Unclear
Li Sheng 2006	Unclear	Low	Unclear	Low	Unclear	Unclear	Low	Unclear	Low	Unclear
Hong Chen 2005	Low	Low	Unclear	Low	Unclear	Unclear	Low	Unclear	Low	Unclear
Qiufeng Tang 2014	Low	Low	Unclear	Low	Unclear	Unclear	Low	Low	Low	Unclear
Zhongqiu Luan 2012	Low	Low	Unclear	Low	Unclear	Unclear	Low	Low	Low	Unclear
Jingjin Yang 2016	Low	Low	Unclear	Low	Unclear	Unclear	Low	Low	Low	Unclear

^a^ Items in agreement with the items in the Cochrane Risk of Bias tool.

### 3.4 Meta-analysis

#### 3.4.1 Meta-analysis of TGF-β1 expression

**FIGURE 3:**
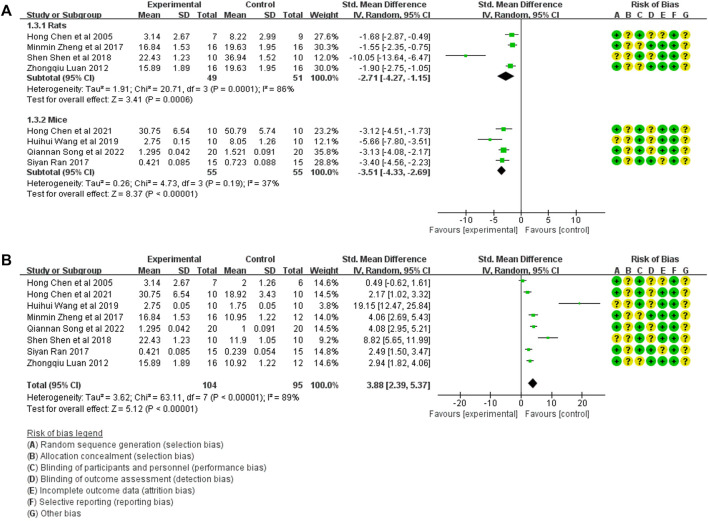
**(A)** Meta-analysis of TGF-β1 expression between treatment group and animal model control group. **(B)** Meta-analysis of TGF-β1 expression between treatment group and blank control group.

The TGF-β1 expression in model control (106 animals) and treatment groups (104 animals) was compared only in eight studies (Chen et al., 2005a; Luan, 2012; Ran, 2017; Zheng et al., 2017; Shen et al., 2018; Wang et al., 2019; Chen et al., 2020; Song et al., 2022a). A heterogeneity test was performed in each study, and I^2^ was 82%. Therefore, the random effect model was used for meta-analysis. In rats, the TGF-β1 expression in the treatment was significantly lower than that in the animal model control group [SMD = −2.71, 95% CI (−4.27, −1.15, *p* < 0.0001]. The I^2^ value was zero after excluding the study by Yu et al. (2018). In mice, TGF-β1 expression in the treatment group was significantly lower than in the animal model control group [SMD = −3.51, 95% CI (−4.33, −2.69), *p* < .0001]. The relevant forest plot is shown in Figure 3A.

The TGF-β1 expression in the treatment group (104 animals) and blank control group (106 animals) was compared in eight studies (Chen et al., 2005a; Luan, 2012; Ran, 2017; Zheng et al., 2017; Shen et al., 2018; Wang et al., 2019; Chen et al., 2020; Song et al., 2022a). A heterogeneity test was performed in each study, and I^2^ was 89%. Therefore, the random effect model was used for meta-analysis. The TGF-β1 expression in the treatment group was significantly higher than that in the blank control group [SMD = 3.88, 95% CI (2.39, 5.37), *p* < .0001], The relevant forest plot is shown in Figure 3B.

#### 3.4.2 Meta-analysis of α-SMA expression

**FIGURE 4:**
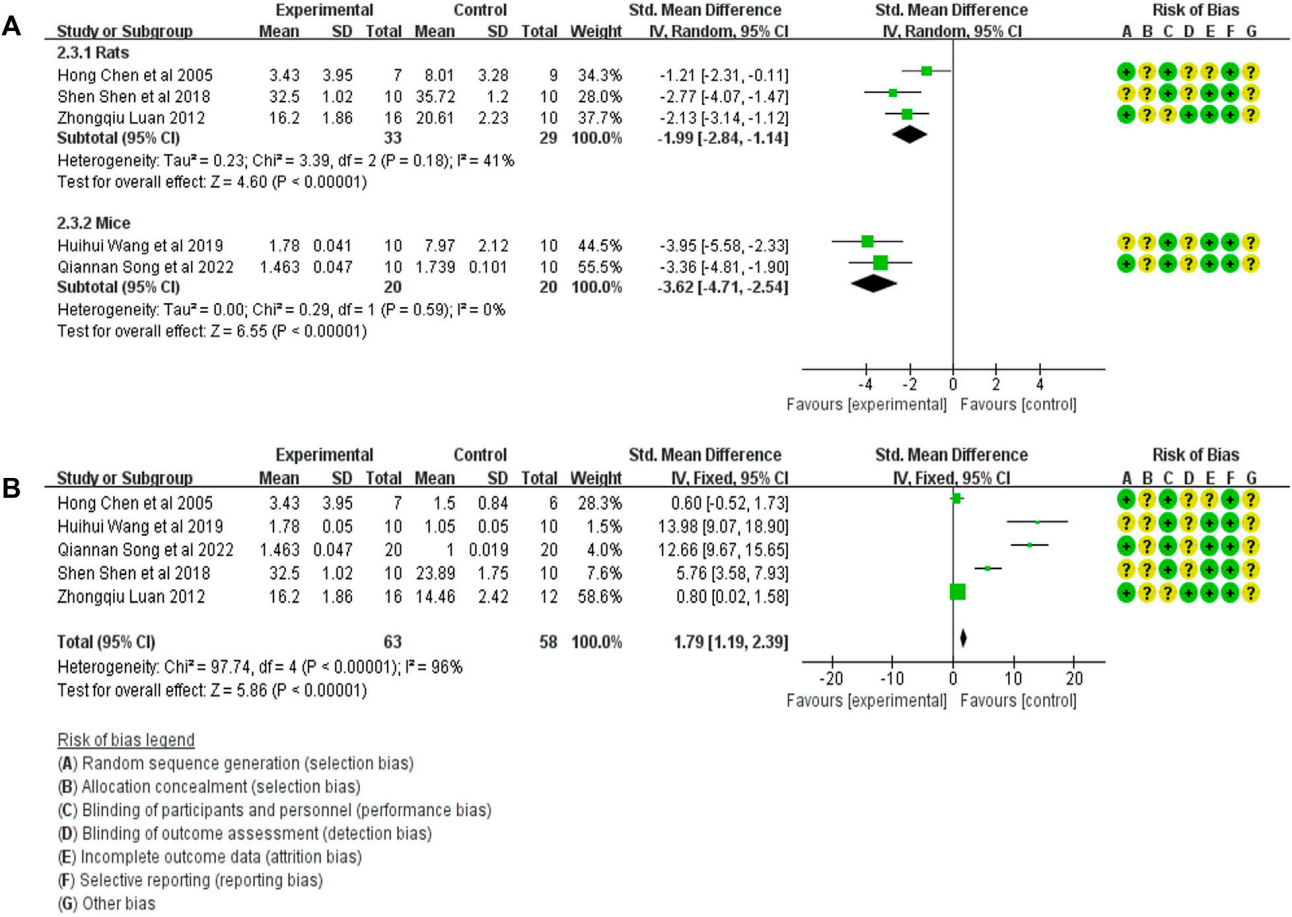
**(A)** Meta-analysis of α-SMA expression between treatment group and animal model control group. **(B)** Meta-analysis of α-SMA expression level between treatment group and blank control group.

Five studies compared α-SMA expression in the treatment group (53 animals) and model control group (55 animals) (Luan, 2012; Shen et al., 2018; Wang et al., 2019; Chen et al., 2020; Song et al., 2022a). A heterogeneity test was performed in each study, and I^2^ was 62%. Therefore, the random effect model was used for Meta-analysis. Due to the heterogeneity of the results obtained by combining all studies is too large, subgroup analysis is conducted to explore the reasons for the large heterogeneity. The study was divided into rats and mice groups, and heterogeneity was found to decrease after grouping. In rats, α-SMA expression in the treatment group was significantly lower than in the animal model control group [SMD = −1.99, 95% CI (−2.84, −1.14), *p* < .0001]. In mice, α-SMA protein in the treatment group was significantly lower than in the animal model control group [SMD = −3.62, 95% CI (−4.71, −2.54), *p* < .0001]. The relevant forest plot is shown in Figure 4A.

Five studies compared the expression of α-SMA in treatment (53 animals) and blank control (55 animals) groups (Luan, 2012; Shen et al., 2018; Wang et al., 2019; Chen et al., 2020; Song et al., 2022a). A heterogeneity test was carried out in each study, and I^2^ was 94%. Therefore, the random effect model was used for Meta-analysis. The results showed that the α-SMA expression in the treatment group was significantly higher than in the blank control group. [SMD = 1.79, 95% CI (1.19, 2.39), *p* < .0001]. The relevant forest plot is shown in Figure 4B.

#### 3.4.3 Meta-analysis of Scr expression

**FIGURE 5:**
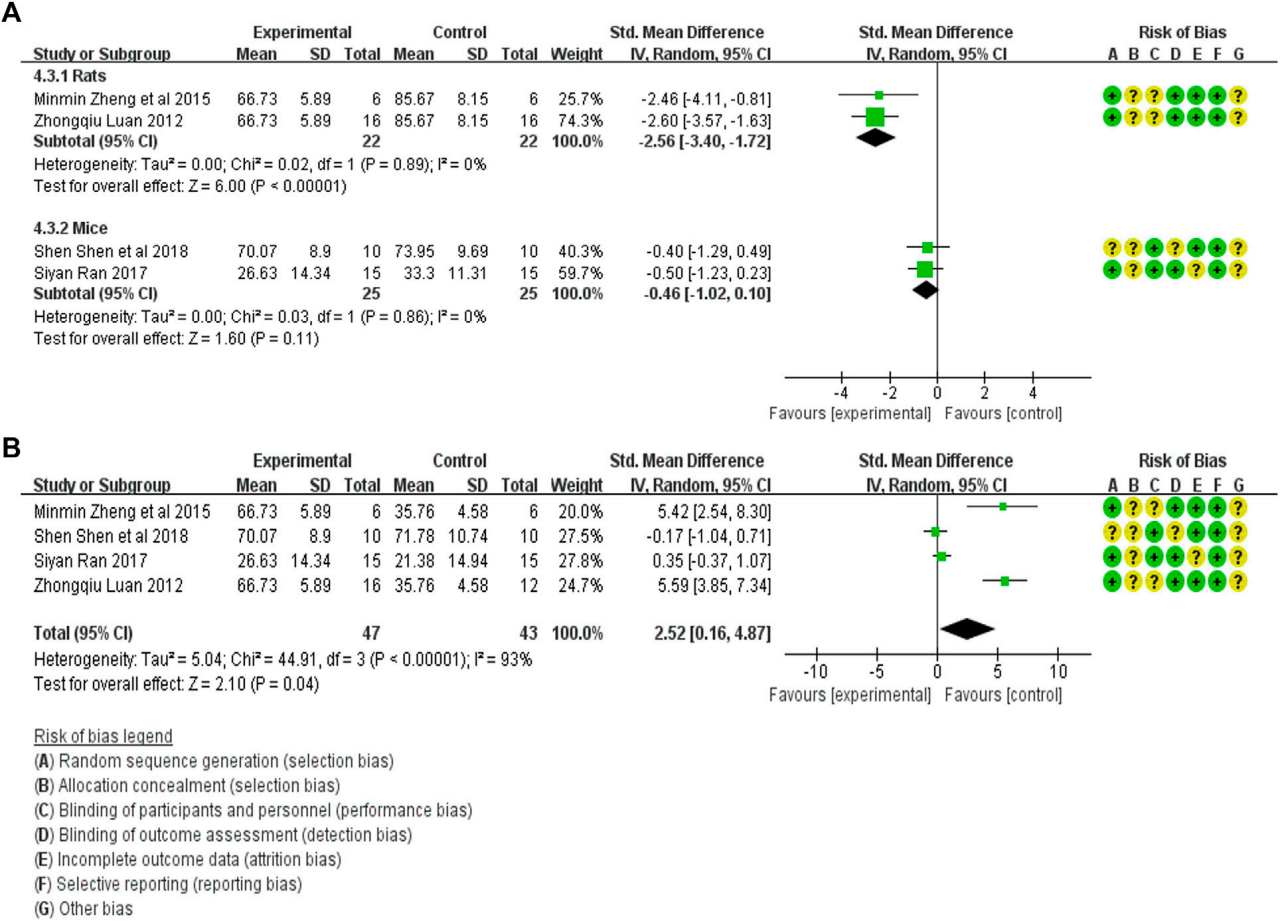
**(A)** Meta-analysis of Scr expression between treatment group and animal model control group. **(B)** Meta-analysis of α-SMA expression level between treatment group and blank control group.

The Scr expression in the treatment group (47 animals) and animal model (47 animals) was compared in four studies (Luan, 2012; Zheng et al., 2015a; Ran, 2017; Shen et al., 2018). A heterogeneity test was performed in each study, and I^2^ was 82%. Therefore, the random effect model was used for meta-analysis. Due to the heterogeneity of the results obtained by combining all studies is too large, subgroup analysis is conducted to explore the reasons for the large heterogeneity. The study was divided into rats and mice groups, and heterogeneity was found to decrease after grouping. In rats, the Scr expression in the treatment group was significantly lower than that in the animal model control group [SMD = −2.56, 95% CI (−3.40, −1.72), *p* < .0001]. In mice, Scr expression in the treatment group was lower than in the animal model control group, but the difference was insignificant. [SMD = −0.46 95% CI (−1.02, 0.10), *p* = .11]. The relevant forest plot is shown in Figure 5A.

The Scr expression in the treatment group (47 animals) and blank control group (43 animals) was compared in four studies (Luan, 2012; Zheng et al., 2015a; Ran, 2017; Shen et al., 2018). A heterogeneity test was carried out in each study and I^2^ was 93%, so a random effect model was used for meta-analysis. The Scr expression in the treatment group was significantly higher than that in the blank control group [SMD = 2.52, 95% CI (0.16, 4.87), *p* < .0001]. The relevant forest plot is shown in Figure 5B.

#### 3.4.4 Meta-analysis of BUN expression

**FIGURE 6:**
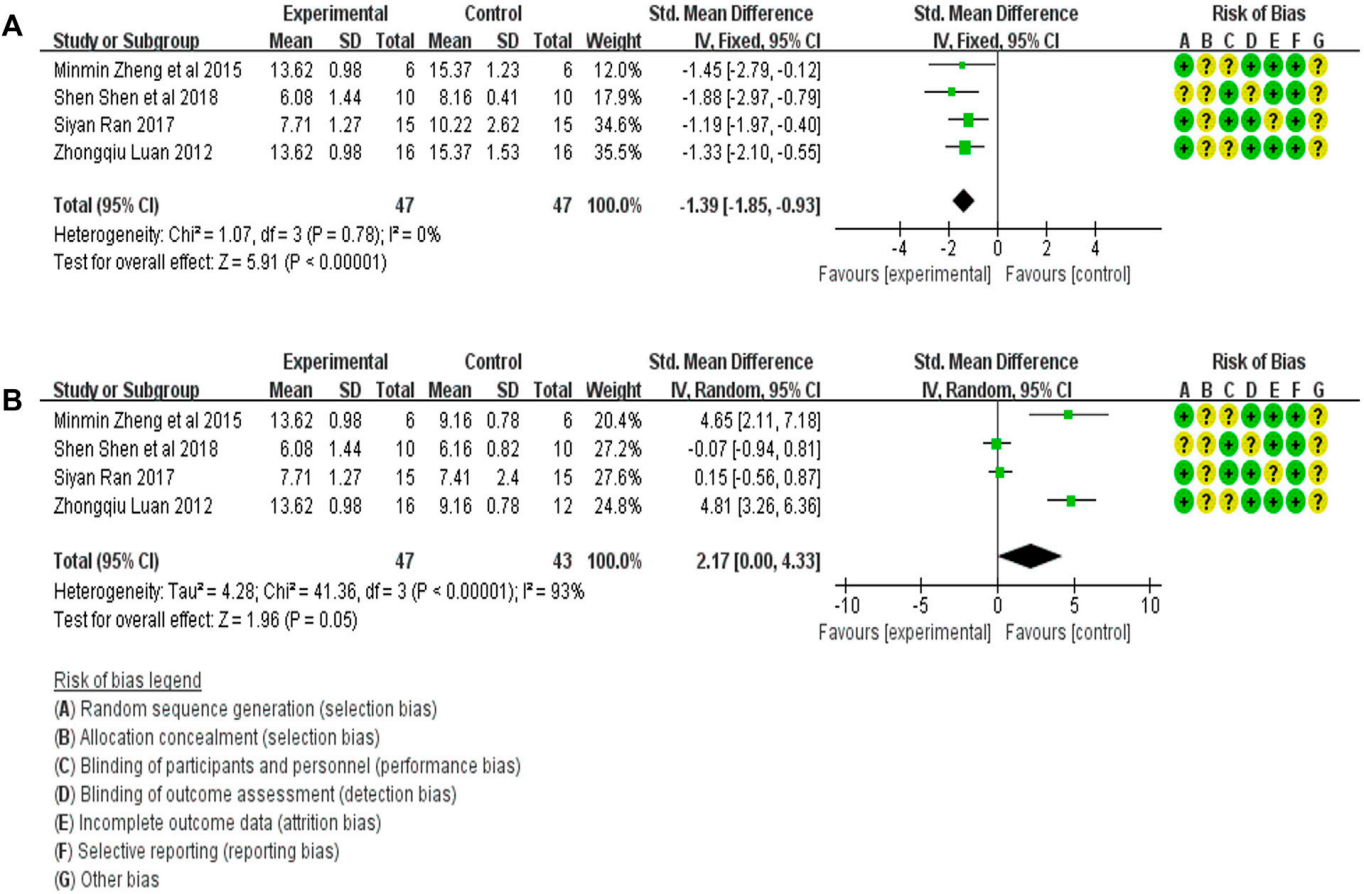
**(A)** Meta-analysis of BUN expression between treatment group and animal model control group. **(B)** Meta-analysis of BUN expression between treatment group and blank control group.

The BUN expression in the treatment (47 animals) and model control (47 animals) groups were compared in four studies (Luan, 2012; Zheng et al., 2015a; Ran, 2017; Shen et al., 2018). A heterogeneity test was carried out in each study, and I^2^ was 0%. Therefore, the fixed effect model was used for meta-analysis. The BUN expression in the treatment group was significantly lower than in the animal model control group [SMD = −1.39, 95% CI (−1.85, −0.93, *p* < .0001]. The relevant forest plot is shown in Figure 6A.

The level of BUN expression in the treatment group (47 animals) and blank control group (43 animals) was compared in four studies (Luan, 2012; Zheng et al., 2015a; Ran, 2017; Shen et al., 2018). A heterogeneity test was carried out in each study, and I^2^ was 93%, so a random effect model was used for Meta-analysis. The BUN expression in the DE group was significantly higher than that in the blank control group [SMD = 2.17, 95% CI (0.00, 4.33), *p* = .05]. The relevant forest plot is shown in Figure 6B.

#### 3.4.5 Meta-analysis of tissue inflammation score

**FIGURE 7:**
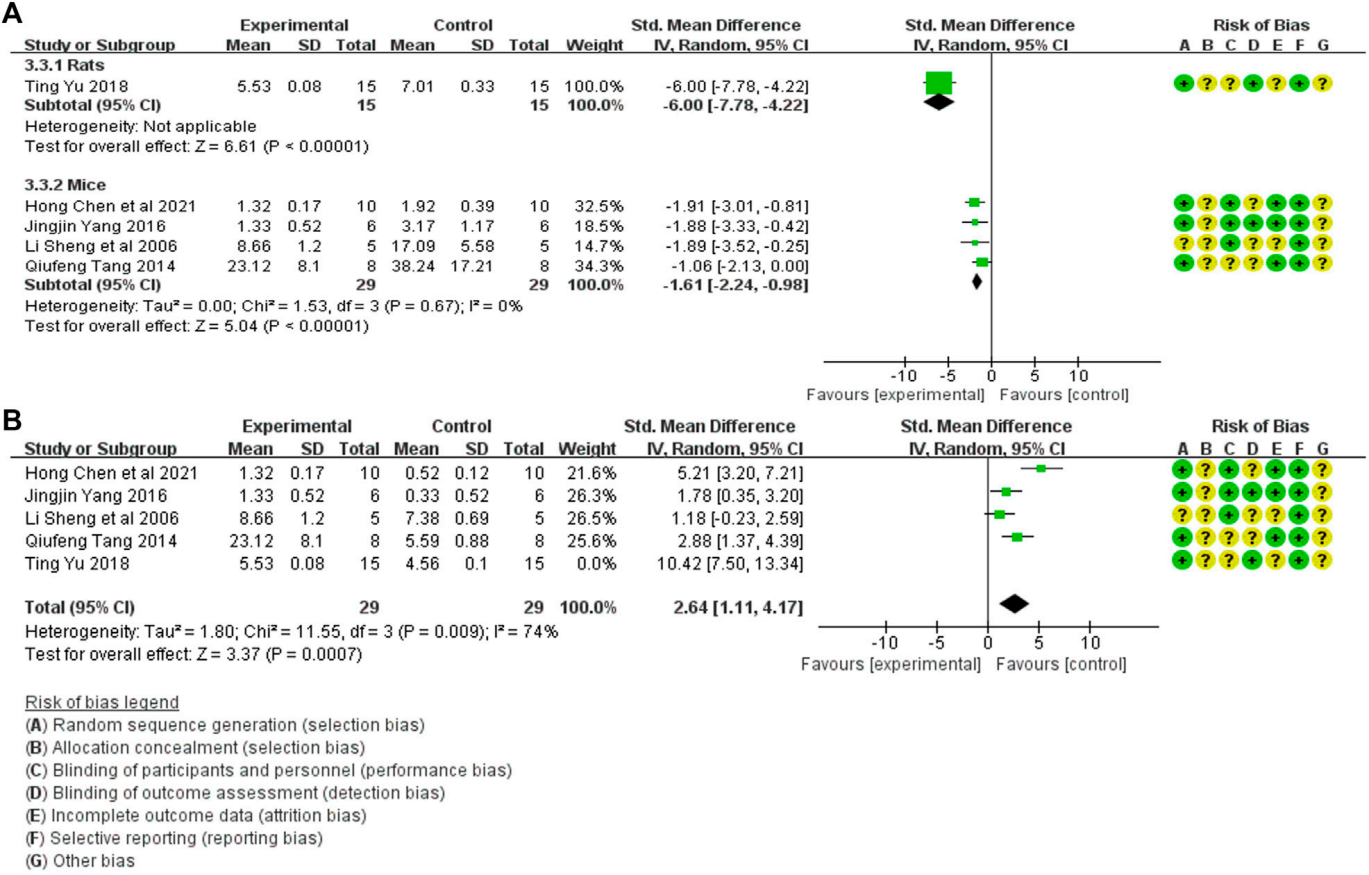
**(A)** Meta-analysis of tissue inflammation score between treatment group and animal model control group. **(B)** Meta-analysis of tissue inflammation score between treatment group and blank control group.

Tissue inflammation scores in the treatment (44 animals) and model control (44 animals) groups were compared in five studies (Sheng et al., 2006; Tang, 2014; Yang et al., 2016; Yu et al., 2018; Chen et al., 2020). A heterogeneity test was conducted in each study, and I^2^ was 90%. Therefore, the random effect model was used for Meta-analysis. Due to the heterogeneity of the results obtained by combining all studies is too large, subgroup analysis is conducted to explore the reasons for the large heterogeneity. The study was divided into rats and mice groups, and heterogeneity was found to decrease after grouping. The rats group was unable to analyze heterogeneity due to the small amount of included studies, but the forest plot indicated that tissue inflammation scores in the treatment group was significantly lower than that in the animal model control group [SMD = −2.56, 95% CI (−3.40, −1.72), *p* < .0001]. In mice, the tissue inflammation scores in the treatment group was lower than in the animal model control group, and the difference was significant. [SMD = −1.61 95% CI (−2.24, −0.98, *p* < 0.0001]. The relevant forest plot is shown in Figure 7A.

Tissue inflammation scores between treatment (44 animals) and blank control (44 animals) groups were mentioned in five studies (Sheng et al., 2006; Tang, 2014; Yang et al., 2016; Yu et al., 2018; Chen et al., 2020). A heterogeneity test was conducted in each study, and I^2^ was 88%. Therefore, the random effect model was used for Meta-analysis. The tissue inflammation scores in the treatment group were significantly higher than in the blank control group. [SMD = 3.25, 95% CI (1.28, 5.22), *p* = .001]. The relevant forest plot is shown in Figure 7B.

### 3.5 Publication bias

**FIGURE 8:**
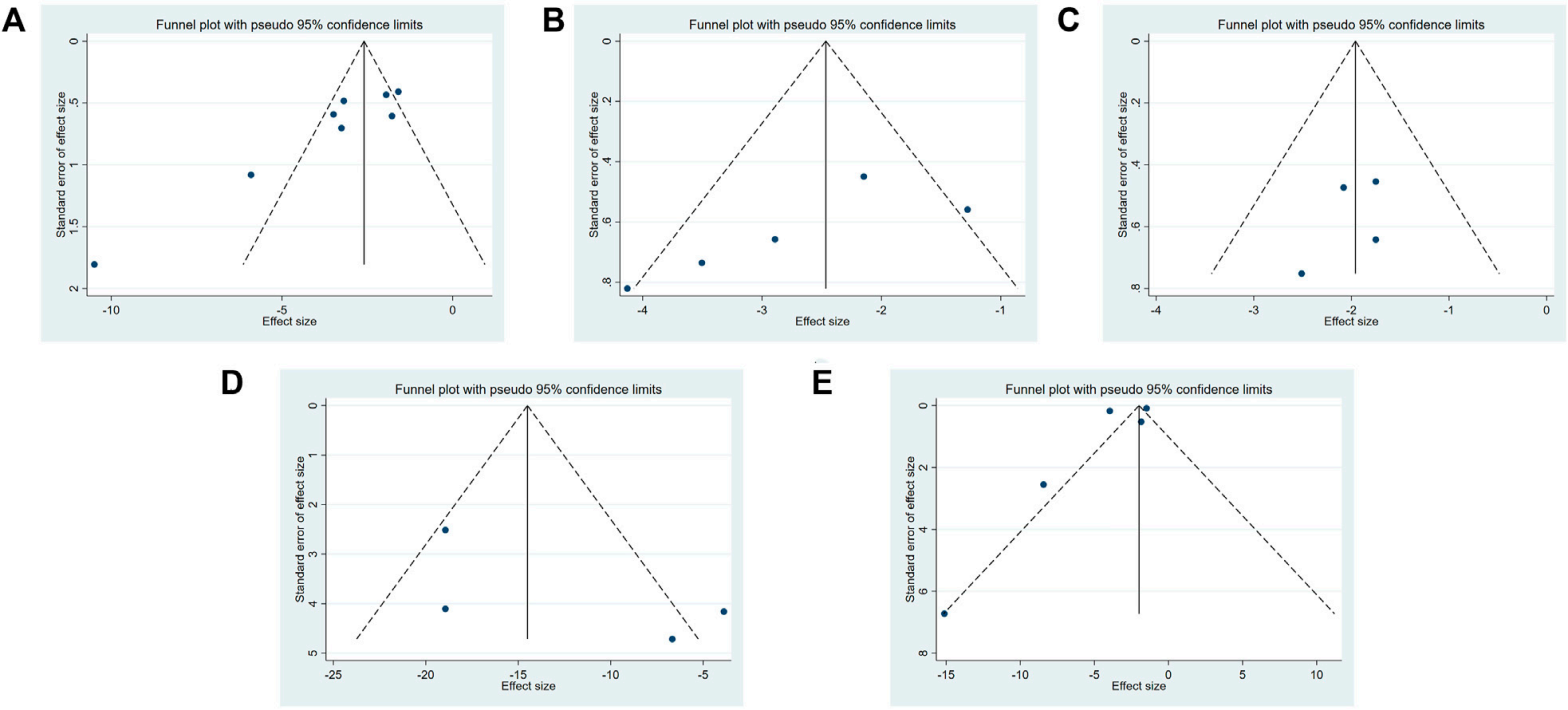
The funnel plot of Dilong extracts for TGF-β1 expression level **(A)**, α-SMA expression level **(B)**, BUN level **(C)**, Scr level **(D)** and tissue inflammation score **(E)**.

Three methods (the funnel plot, the Egger’s test, and the Begg’s test) for assessing the publication bias. A funnel plot for each indicator was drawn using Stata 16.0. software. A funnel plot was drawn for each indicator and is shown in Figure 8. Publication bias (Egger’s test: *p* = .003 < .05 and Begg’s test: *p* = .019 < .05) was found in TGF-β1 protein expression. No evidence of publication bias was found for the remaining biomarkers (α-SMA: Egger’s test: *p* = .127 and Begg’s test: *p* = .086; Scr: Egger’s test: *p* = .279 and Begg’s test: *p* = .308; BUN: Egger’s test: *p* = .463 and Begg’s test: *p* = .089; the degree of tissue inflammation: Egger’s test: *p* = .444 and Begg’s test: *p* = .806).

## 4 Discussion

Thirteen studies published in Chinese and English languages were included in this study. The intervention effect of DE on tissue fibrosis was compared with the blank and model control groups. The meta-analysis results confirmed that DE effectively inhibited the expression of TGF-β1, α-SMA, Scr and BUN and reduced tissue inflammation. The heterogeneity index is high (I^2^ > 50), and thus subgroup (rats and mice) analysis was conducted to identify the reasons for high heterogeneity. In the rats, the TGF-β1 protein expression from the forest plot the heterogeneity (I^2^) is 86%, and the *p* value is .0006. The heterogeneity value is zero after excluding the study of Shen et al. (2018). This finding may be because this study used lumbrokinase for therapeutic intervention. In contrast, in other studies, the therapeutic intervention is Dilong decoction. The low heterogeneity (I^2^ = 37%) in the mice suggests that these results are plausible. The subgroup analysis on the remaining four indicators showed low heterogeneity, indicating that earthworm extract has a beneficial effect in reducing tissue fibrosis. The greater heterogeneity in the outcome indicator of tissue inflammation score may be due to the different animal models used in the experiments. After removing the experimental data of Yu et al. (2018), only the experimental data of mice as experimental models were analyzed, and the heterogeneity was reduced to 0.

Among all the outcome indicators, the level of the treatment group was lower than that of the animal model control group, but higher than that of the blank control group. This outcome may indicate that the earthworm extract did attenuate the fibrosis process of the animals with tissue fibrosis, but the reversal of fibrosis was not completed within the time reported in the study. Compared with the normal animals without modeling, albeit slight, there was also a certain degree of tissue fibrosis (because compared with the blank group, that is, the animal group without modeling, the outcome was beneficial to the blank group).

Renal interstitial fibrosis is a common pathological feature of various renal diseases that develop into end-stage renal disease. Its mechanism is mainly related to the proliferation of interstitial fibroblasts and the excessive accumulation of extracellular matrix. TGF-β1 is generally regarded as an inhibitor of excessive inflammation. It also accumulates collagen, which progresses to tissue fibrosis (Kim et al., 2017; Lodyga and Hinz, 2019). Myofibroblasts are the key pathogenic cells of fibrotic diseases, mainly derived from EMT, closely related to the increase of α-SMA expression. In addition to fibrosis-related proteins, the serum biochemical indicators (BUN and Scr) were also evaluated to determine the efficacy of DE in renal fibrosis. The earthworm extract could protect renal function, reduce renal tissue damage, inhibit excessive activation of fibroblasts, and downregulate the expression of TGF-β1 and α-SMA. The effect of the low-dose group of earthworm components was better and delayed the occurrence and development of renal interstitial fibrosis.

The mechanism of earthworm extract attenuates fibrosis process also needs to be further explored. Li et al. (2022) found that the purified protein (P2) isolated from *P. aspergillum* showed great regulatory effect on TGF-β/Smad pathway in MRC-5 cells induced by TGF-β1, a finding that provides a theoretical basis for its clinical application in pulmonary fibrosis. In addition to downregulating the expression of tissue fibrosis-related proteins, some studies have also attempted to investigate the real reasons for reducing fibrosis from other perspectives. Peluso et al. (2015) found that workers with lung fibrosis due to long-term exposure to SiO_2_ also had rapid oxidative DNA damage in the nasal lining epithelial cells. In addition, Yang et al. (2016) results also pointed out that after intragastric administration of earthworm extract, the malonaldehyde (MDA) level in serum of mice was significantly reduced, and the activity of superoxide dismutase (SOD) antioxidant enzymes related to oxidative stress was also increased. It is speculated that earthworm extract may slow down the fibrosis process by increasing the capacity of antioxidant system. In addition, it is worth pondering that for earthworms, the more well-known pharmacological activity is its fibrinolytic activity. Perhaps the mechanism of reducing fibrosis is related to the fibrinolytic activity and anticoagulant effect of earthworm fibrinolytic enzyme.

Some studies have analysed other biomarkers not included in this meta-analysis. These indicators are related to inflammatory factors, and inflammatory cell proliferation rate carried out in rats, mice and cells (Table 3). In addition to the research on lung, liver and kidney tissues mentioned in the article, the researchers also applied the earthworm extract to reverse the process of cardiomyoblast cell fibrosis. Huang et al. (2019) induced H9c2 cells under high HCl conditions. After induction, the levels of fibrosis-related mediators in the cells were up-regulated, and after treatment with earthworms, cell activation-related pathways were activated, suggesting that earthworms have good cardiac protection. Lai et al. (2014) established a rat cardiac fibrosis model with second-hand smoke as an inducer. The results indicated that after treatment with earthworm, the process of cardiac fibrosis slowed down and had a certain protective effect on the heart.

**TABLE 3:** Characteristics of Dilong extract for reducing fibrosis.

Studies	Characteristics	Results		Materials	Sampling time	References
		Intervention	Control			
Qiannan Song 2022	α-SMA mRNA	0.398 ± 0.059	0.571 ± 0.061	Mice	5 weeks	Song et al. (2022)
	TGF-β1 mRNA	0.392 ± 0.065	0.458 ± 0.042	Mice	5 weeks	Song et al. (2022)
Chaohung Lai 2014	IVSd (mm)	1.47 ± 0.6	1.18 ± 0.03	Rats	1 month	Lai et al. (2014)
	LVPWd (mm)	1.50 ± 0.10	1.16 ± 0.09	Rats	1 month	Lai et al. (2014)
	EF (%)	76.16 ± 2.87	74.18 ± 2.24	Rats	1 month	Lai et al. (2014)
	FS (%)	40.59 ± 2.86	38.62 ± 1.86	Rats	1 month	Lai et al. (2014)
Minmin Zheng 2015	BMP-7	17.76 ± 1.05	14.66 ± 1.01	Rats	4 weeks	Zheng et al. (2015b)
	ALK-2	0.43 ± 0.02	0.16 ± 0.03	Rats	4 weeks	Zheng et al. (2015b)
	P-Smad5	0.35 ± 0.02	0.08 ± 0.01	Rats	4 weeks	Zheng et al. (2015b)
Hong Chen 2020	IL-6	17.48 ± 2.09	24.38 ± 4.01	Mice	4 weeks	Chen et al. (2020)
	IL-17	25.27 ± 5.03	89.47 ± 8.83	Mice	4 weeks	Chen et al. (2020)
Hong Chen 2005	uPA	3.43 ± 2.23	7.67 ± 4.09	Rats	8 weeks	Chen et al. (2005a)
	PAI^−1^	3.14 ± 2.67	7.11 ± 3.86	Rats	8 weeks	Chen et al. (2005a)
Zhongqiu Luan 2012	Col-Ⅳ	13.28 ± 1.23	17.13 ± 1.89	Rats	4 weeks	Luan (2012)
	NF-kβ	0.27 ± 0.03	0.39 ± 0.04	Rats	4 weeks	Luan (2012)
	P38MAPK	0.32 ± 0.03	0.60 ± 0.06	Rats	4 weeks	Luan (2012)

*IVSd, interventricular septal end diastolic dimension; LVPWd, left ventricular end diastolic posterior wall dimension; EF, ejection fraction; FS, fraction shortening.

There are a few limitations to this study. 1) Only a few studies were included in this study, and the small size might have influenced this study’s outcome. 2) A significant publication bias was observed in the studies of TGF-β1 expression. 3) The method of extraction (some used water and some used ethanol) is not uniform in all the studies. 4) The exact chemical composition of DE is not known. 5) Differences in intervention methods, one study used lumbrokinase, and others used the extract. 6) Most studies on the antifibrotic effects of earthworms are from China. More studies should be conducted to confirm the effectiveness, beyond any element of doubt, of earthworms in tissue fibrosis.

## 5 Conclusion

This study analysed 325 studies from five scientific databases, and 14 were included in the meta-analysis. The earthworms can reduce the expression of fibrosis-related proteins and the degree of tissue inflammation, inhibit the tissue fibrosis process, and play an important role in alleviating lung fibrosis, myocardial fibrosis, renal interstitial fibrosis, liver fibrosis, etc. According to biochemical and histomorphological experiments, the extract can delay the process of renal interstitial fibrosis, inhibit the proliferation of lung fibroblasts and the rate of fibrosis of lung tissue cells, accelerate the deposition and degradation of extracellular matrix, and effectively reduce the damage caused by liver fibrosis and the degree of liver fibrosis to delay liver fibrosis. Dilong has great potential for future research to cure pulmonary fibrosis caused by a novel coronavirus. Currently, some preparations contain earthworms, mainly for treating cardiovascular and cerebrovascular diseases, as well as cough and asthma. Future research studies should concentrate on identifying the bioactive components and preparation of the most active extracts to widen the therapeutic prospects of earthworms. (Chen et al., 2005b; Zheng et al., 2015b).

## Data Availability

The original contributions presented in the study are included in the article/Supplementary Material, further inquiries can be directed to the corresponding authors.

## Supplementary material

**Supplementary Material S1** | Search strategy.

**Supplementary Material S2** | PRISMA 2020 Checklist.

**Supplementary Material S3** | AMSTAR 2 Checklist.

**Supplementary Material S4** | Original data.

**Supplementary Material S5** | Not included articles.
