# Supplementary material for: Effect of Pheretima aspergillum on reducing fibrosis: A systematic review and meta-analysis
Source: Front Pharmacol. 2022 Dec 23;13:1039553. doi: 10.3389/fphar.2022.1039553 (PMC9816480; doi:10.3389/fphar.2022.1039553)

Supplementary Material S1-Search Strategy

Pubmed:12

(((((fibrosis[Title/Abstract]) OR (anti-fibrosis[Title/Abstract])) OR (pulmonary fibrosis[Title/Abstract])) OR (liver fibrosis[Title/Abstract])) OR (hepatic fibrosis[Title/Abstract])) AND ((((((((((((((((((((Oligochaeta[Title/Abstract]) OR (Oligochaetas[Title/Abstract])) OR (Dilong[Title/Abstract])) OR (Di Long[Title/Abstract])) OR (Earthworms[Title/Abstract])) OR (Earthworm[Title/Abstract])) OR (Lumbricus terrestris[Title/Abstract])) OR (Lumbricus terrestri[Title/Abstract])) OR (terrestris, Lumbricus[Title/Abstract])) OR (Lumbricus[Title/Abstract])) OR (Eisenia worm[Title/Abstract])) OR (Eisenia worms[Title/Abstract])) OR (worm, Eisenia[Title/Abstract])) OR (Eisenia foetida[Title/Abstract])) OR (Eisenia foetidas[Title/Abstract])) OR (foetidas, Eisenia[Title/Abstract])) OR (Eisenia fetida[Title/Abstract])) OR (Eisenia fetidas[Title/Abstract])) OR (fetida, Eisenia[Title/Abstract])) OR ("Oligochaeta"[Mesh]))

Web of Science：45

TS=( Oligochaeta OR Oligochaetas OR Dilong OR Di Long OR Earthworms OR Earthworm OR Lumbricus terrestris OR Lumbricus terrestri OR terrestris, Lumbricus OR Lumbricus OR Eisenia worm OR Eisenia worms OR worm, Eisenia OR Eisenia foetida OR Eisenia foetidas OR foetidas, Eisenia OR Eisenia fetida OR Eisenia fetidas OR fetida, Eisenia)

TS=( fibrosis OR anti-fibrosis OR pulmonary fibrosis OR liver fibrosis OR hepatic fibrosis)


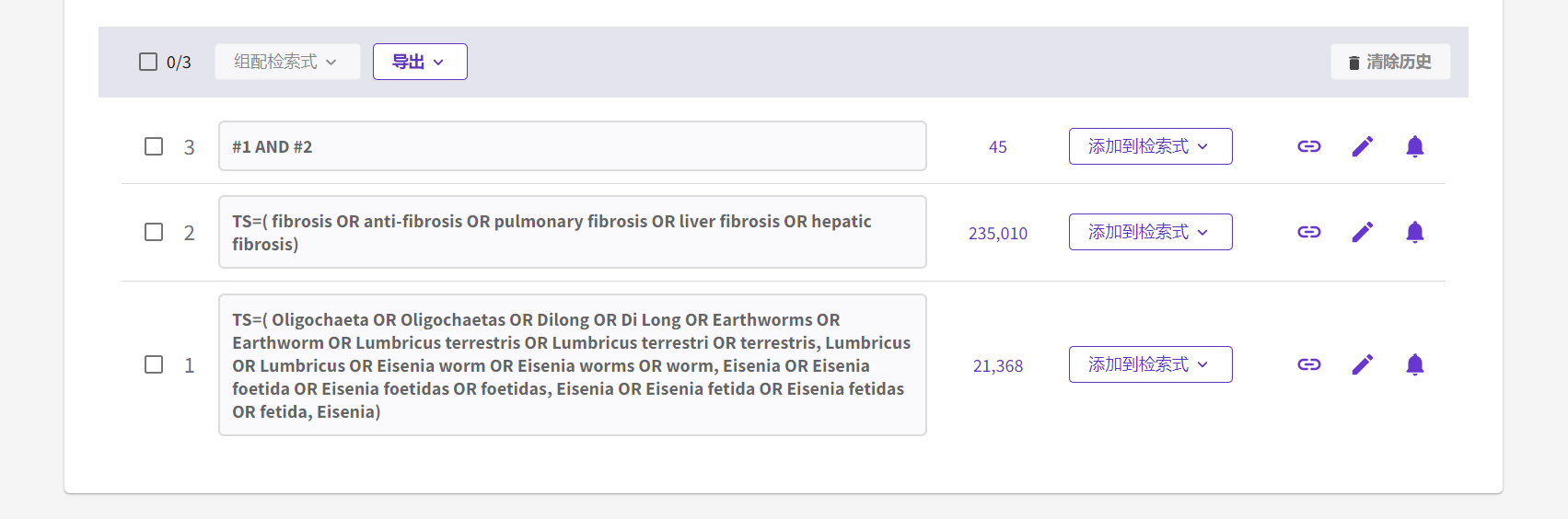


VIP：51


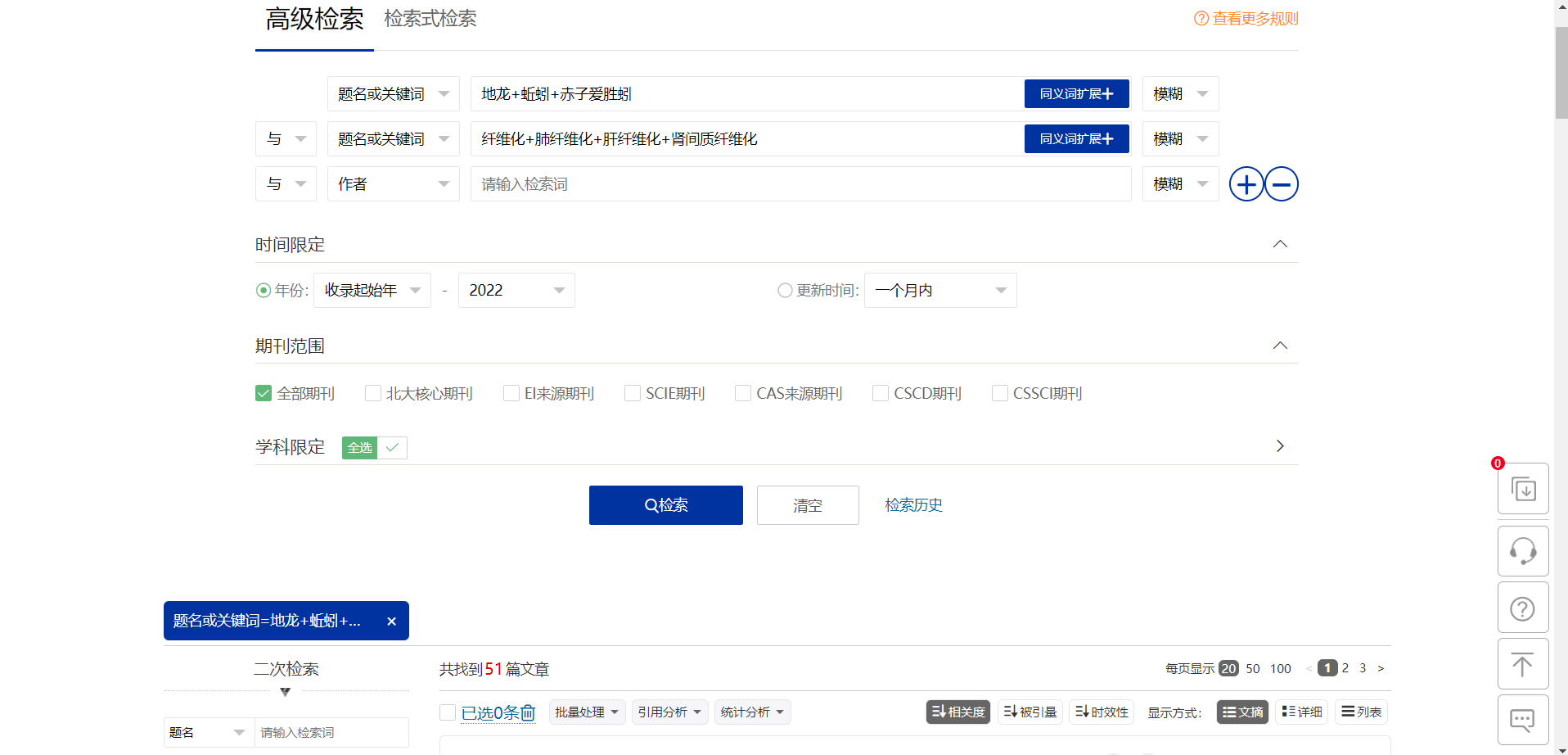


WANFANG:119


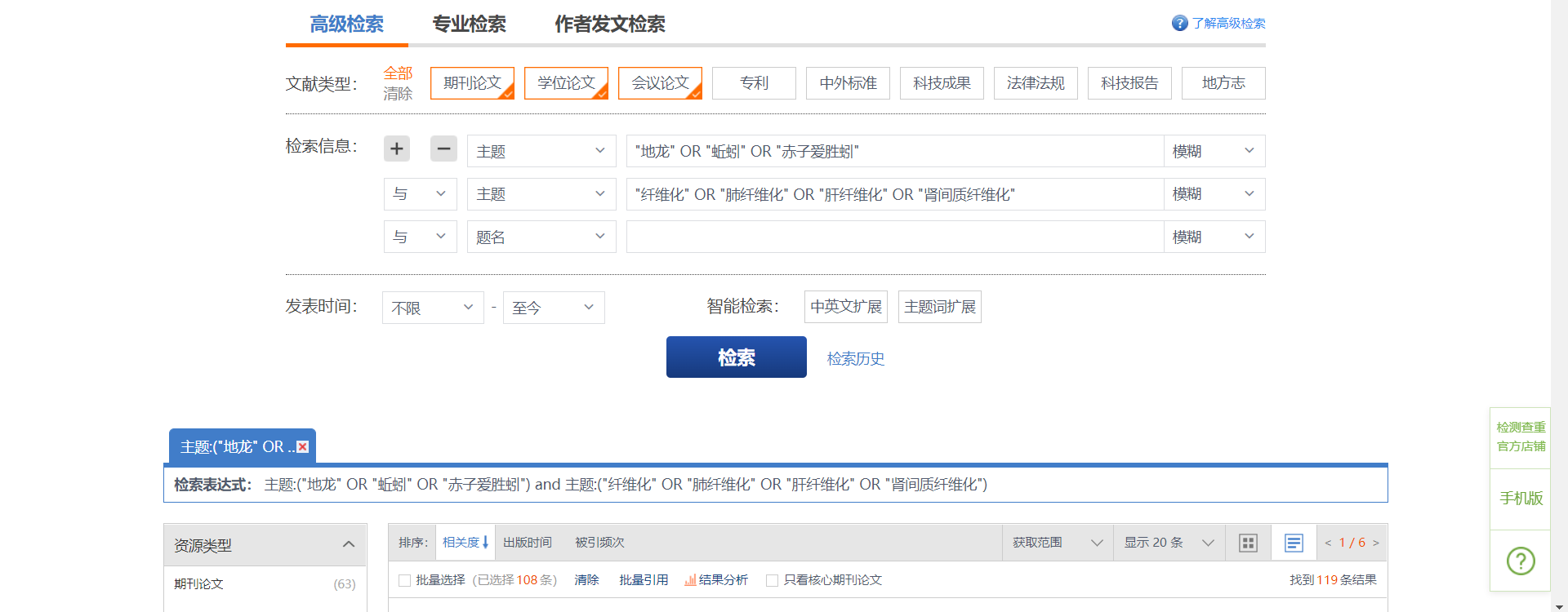


CNKI：98


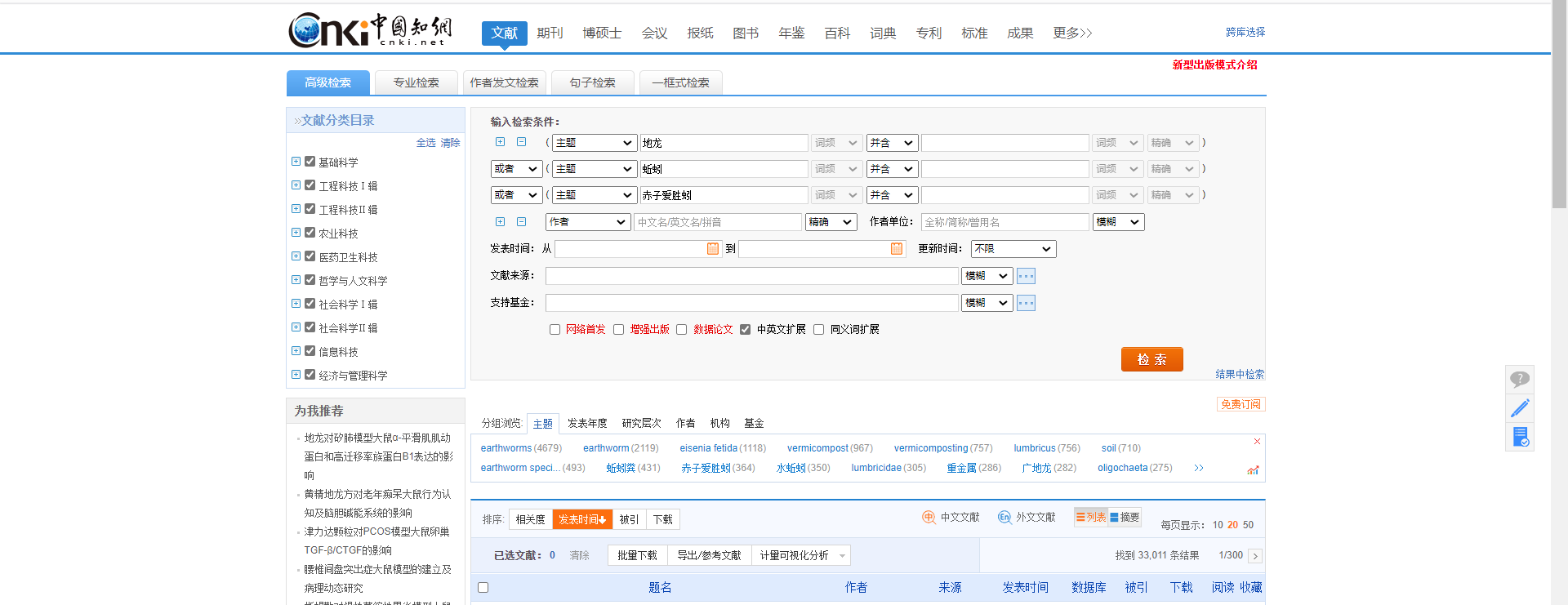


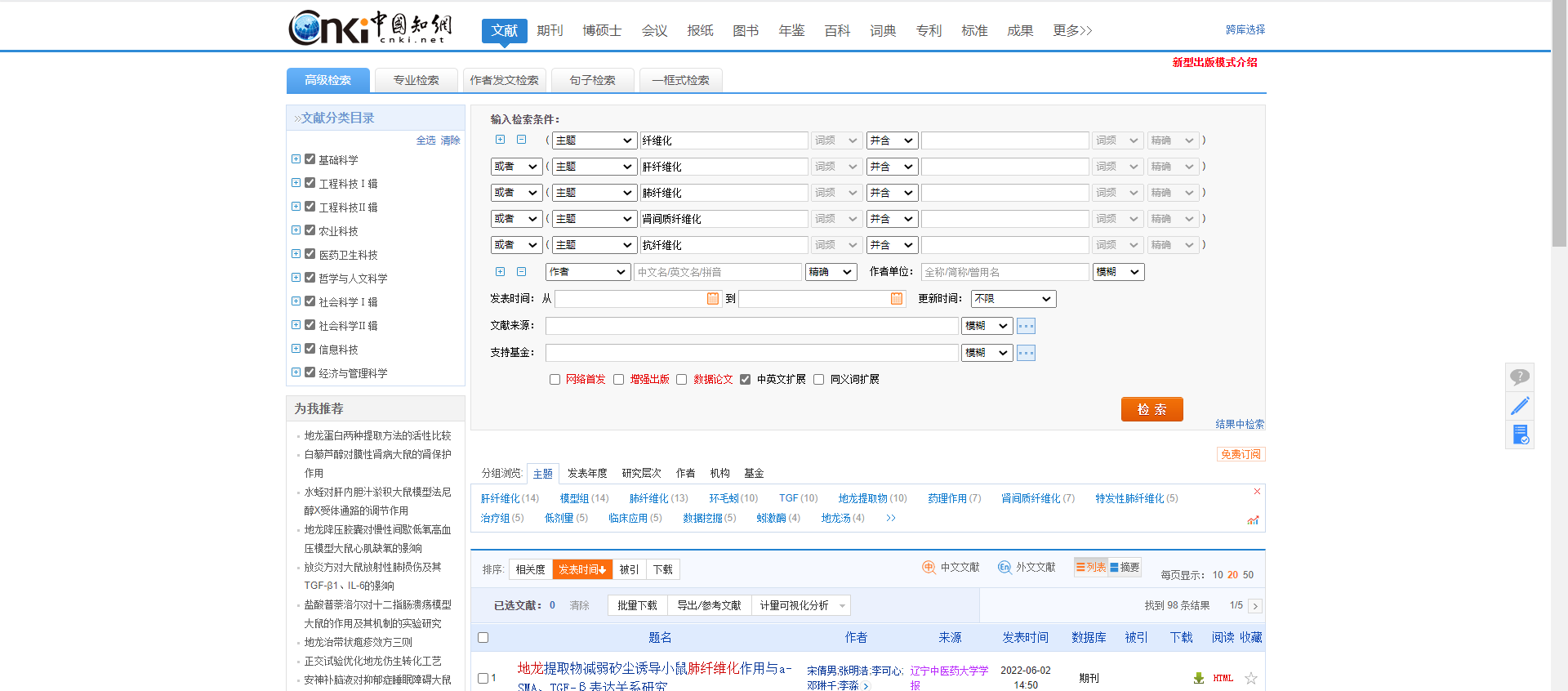


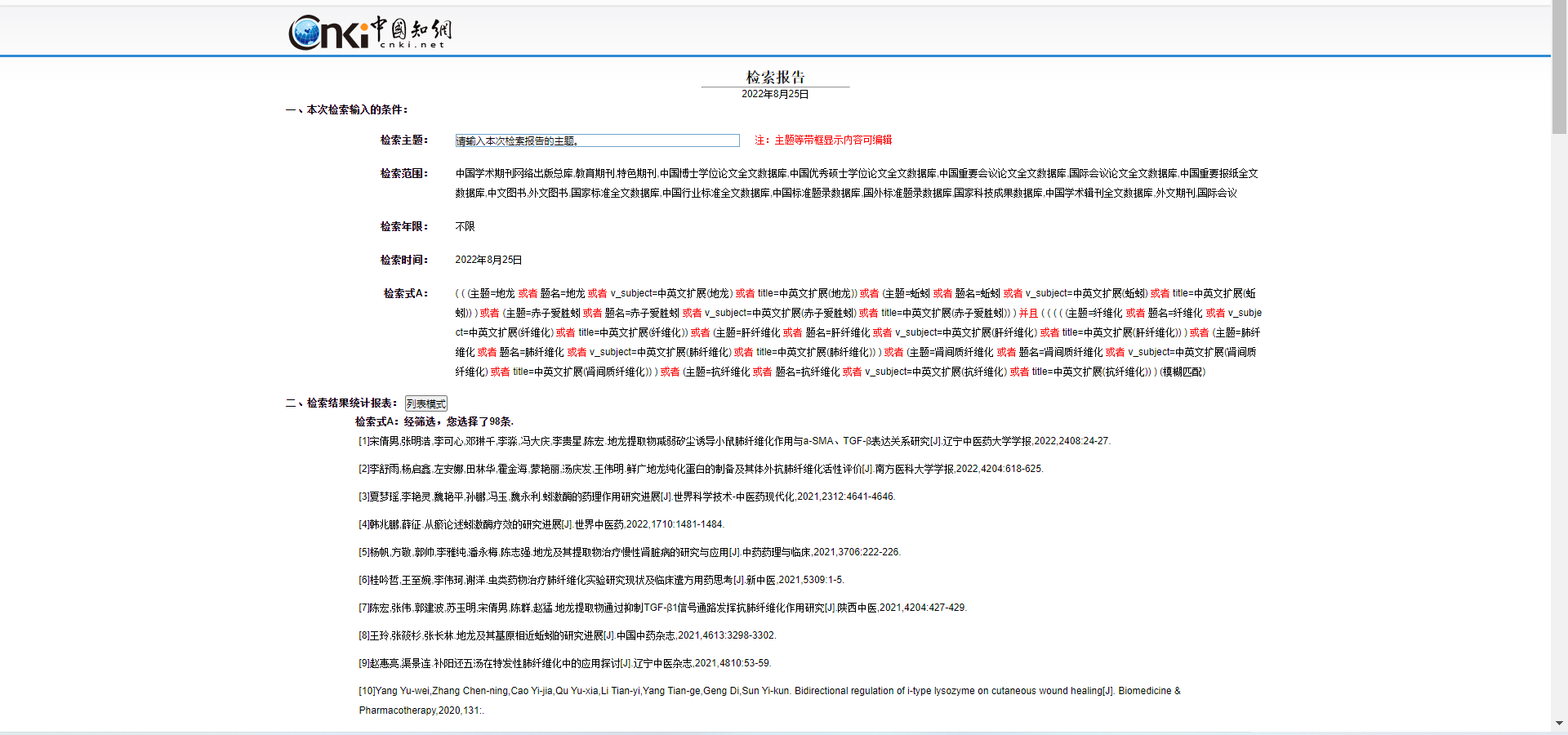


SinoMed:0


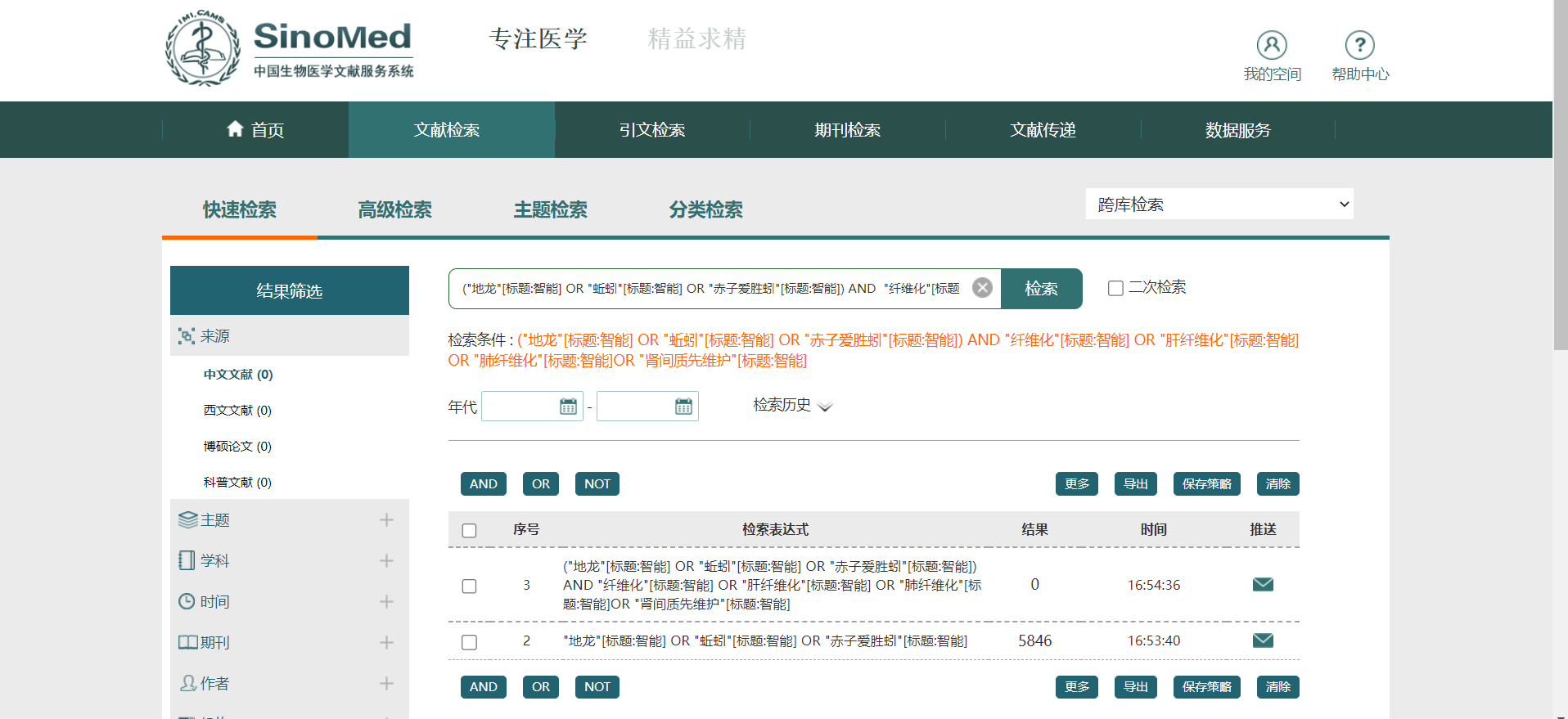

Supplement: Supplementary file 2 [file DataSheet3.DOCX]
