# Supplementary material for: Effect of Pheretima aspergillum on reducing fibrosis: A systematic review and meta-analysis
Source: Front Pharmacol. 2022 Dec 23;13:1039553. doi: 10.3389/fphar.2022.1039553 (PMC9816480; doi:10.3389/fphar.2022.1039553)
Supplement: Supplementary file 3 [file DataSheet5.DOCX]

Duplicated Data:

[1]Chen, H. Lu, Y. Q., et al.(2005) Effects of earthworm 2 on the expressions of α-SMA、TGFβ1、uPA and PAI-1 in hepatic fibrosis rat. Jiangsu Medical Journal, 2005(06):443-445+486.DOI:10.19460/j.cnki.0253-3685.2005.06.016. (In Chinese)

[2] Chen, H. Lu, Y. Q., et al.(2005) Effects of Dilong No. 2 on Hepatic Stellate Cell Activation and TGF-β1 Protein Expression in Hepatic Fibrosis Model Rats. Jiangsu Journal of Traditional Chinese Medicine,2005(01):50-52. (In Chinese)

[3] Chen, H. Lu, Y. Q., et al.(2004) Effects of Dilong No. 2 on mRNA and Protein Expression of TGF-β1, MMP-13 and TIMP-1 in Rats with Hepatic Fibrosis. World Chinese Journal of Digestology,2004(10):71-75. (In Chinese)

[4]Lu, Y. Q., Liu, S.Y., et al.(2004) The study of Earthworm Ⅱ in preventing rat hepatic fibrosis induced by CC14 . Chinese Journal of Gastroenterology and Hepatology,2004(03):225-227. (In Chinese)

[5]Lu, Y. Q. Basic study of Dilong 2 inhibiting experimental hepatic fibrosis in rats. Southeast University,2004. (In Chinese)

[6]Zhao, J., Pan, R., et al.(2007) Eisenia fetida Protease-III-1 Functions in Both Fibrinolysis and Fibrogenesis. Journal of Biomedicine and Biotechnology, 2007, 2007.

[7]Zhao, J., Pan, R., et al.(2007) Eisenia fetida Protease-III-1 Functions in Both Fibrinolysis and Fibrogenesis. Journal of Biomedicine and Biotechnology, 2007, 2007.

Irrelevant to study content:

[1]Zhao, H. L., Qu, J. L.(2021)Discussion on Application of Buyang Huanwu Decoctionin Idiopathic Pulmonary Fibrosis. Liaoning Journal of Traditional Chinese Medicine,2021,48(10):53-59.DOI:10.13192/j.issn.1000-1719.2021.10.015. (In Chinese)

[2]Yang, Y. W., Zhang, C. N., Cao, Y. J., et al.(2020) Bidirectional regulation of i-type lysozyme on cutaneous wound healing. Biomedicine &amp; Pharmacotherapy,2020,131.

[3]Ji, L. M. Effects of Buyang Huanwu Decoction on renal interstitial fibrosis in rats. Abstracts of the 5th World Integrative Medicine Congress 2017:54.DOI:10.26914/c.cnkihy.2017.000387. (In Chinese)

[4] Environmental Toxicology; New Environmental Toxicology Study Findings Recently Were Reported by Researchers at China Medical University and Hospital (Lumbrokinase from earthworm extract ameliorates second-hand smoke-induced cardiac fibrosis)[J]. Ecology Environment &amp; Conservation,2015.

[5]Song, W. S.(2015) Pharmacodynamics study on anti-asthmatic earthworm compound. Guangdong Pharmaceutical University. (In Chinese)

[6]Bao, H., et al.(2012) Effect of Geosaurus and Zaocys on Inducible Nitric Oxide Synthase and Endothelin-1 in Rats with Mesangio Proliferative Glomerulonephritis. World Journal of Integrated Traditional and Western Medicine,7(12):1034-1037.DOI:10.13935/j.cnki.sjzx.2012.12.020. (In Chinese)

[7]Chen, B.H.(2013) Clinical observation of Bu Yang Huan Wu Tang treatment of chronicobstructive pulmonary disease with pulmonary fibrosis. Chengdu University of TCM. (In Chinese)

[8]Chen, Y. J.(2010) Study on effects of Traditional Chinese Drug Dilong on Airway Remodeling in Experimental Rat Model of Asthma. Shandong University of TCM. (In Chinese)

[9]Ge, N. (2010)Protection and Mechanism Research of Earthworm and Lumbrokinase on Diabetic Kidney disease. Guangzhou University of TCM. (In Chinese)

[10]Yang, Y. N. (2008)The inhibitory action of Earthworm to airway remodeling in asthma. Shandong University. (In Chinese)

[11]Li, Q. C., et al. (2007) Experimental Study on the Effect of Dilong Huoxue Decoction on Adriamycin Nephrosclerosis. Journal of Clinical Research (09):1508-1511. (In Chinese)

[12]Zhang, X. Y. (2006)Three Cases of Respiratory System. Compilation of Papers of the 23rd National Symposium on Pediatrics of Traditional Chinese Medicine & Workshop of Famous Pediatrician 227-229. (In Chinese)

[13]Wang, S., Liang R. (2004)Study on the correlation between the pharmacology of Chinese medicine earthworm and airway remodeling in asthma. Journal of Guangdong Pharmaceutical University (01):60-62.DOI:10.16809/j.cnki.1006-8783.2004.01.023. (In Chinese)

[14]Hao, C.(1984)Effect and Bioavailability of Rifampin in Reducing Prednisolone. Medical Journal of National Defending Forces in Northwest China (03):268-269. (In Chinese)

[15]Lin, Y. H. (1984)Treatment of idiopathic pulmonary fibrosis. Chinese Journal of Postgraduates of Medicine ,(05):7-8. (In Chinese)

Not contain useful indicators:

[1]Li, S.Y., Yang, Q.X., et al. (2022) Preparation of purified proteins from fresh Pheretima and their inhibitory effect against pulmonary fibrosis in mic. Journal of Southern Medical University, 42(04):618-625. (In Chinese)

[2]Qiu, Y.Y. Effects of earthworm ultrafiltration on cardiac function and myocardial fibrosis in SHR rats and its mechanis. Gansu University of TCM 2020-07-01. (In Chinese)

[3]Huang, P. C., et al.(2019) Pheretima aspergillum extract attenuates high-KCl-induced mitochondrial injury and pro-fibrotic events in cardiomyoblast cells. Environmental toxicology,34(8).

[4]Zhao, X. K. (2018)The clinical and experimental study of intervention bu filong jiangya capsule from Rho/ROCK pathway on hypertensive left ventricular hypertrophy. [D].Lanzhou University. (In Chinese)

[5]Chai, H. (2018) Effect of Dilongjiangya capsule on early renal damage in spontaneously hypertensive rats based on AngⅡ-TLR4/NF-κB pathway. Gansu University of TCM. (In Chinese)

[6]Li, S. (2017)Antioxidant effect of selenium-enriched earthworm on chronic liver fibrosis mice induced by CCl_4, Hubei University for Nationalities. (In Chinese)

[7]Yang, J. J.,Wang, T., et al. (2016) Earthworm extract attenuates silica-induced pulmonary fibrosis through Nrf2-dependent mechanisms. Laboratory investigation; a journal of technical methods and pathology, 96(12).

[8]Han, C. K., et al. (2014)Dilong prevents the high-KCl cardioplegic solution administration-induced apoptosis in H9c2 cardiomyoblast cells mediated by MEK. The American journal of Chinese medicine,42(6).
