# Supplementary material for: Effect of Pheretima aspergillum on reducing fibrosis: A systematic review and meta-analysis
Source: Front Pharmacol. 2022 Dec 23;13:1039553. doi: 10.3389/fphar.2022.1039553 (PMC9816480; doi:10.3389/fphar.2022.1039553)
Supplement: Supplementary file 5 [file DataSheet2.DOCX]

**S-Table1. Data of TGF-β1 expression extracted from included studies between treatment group and animal model control group.**

| Study or Subgroup | Experimental | | total | Control | | total |
| --- | --- | --- | --- | --- | --- | --- |
|  | mean | SD |  | mean | SD |  |
| Rats |  |  |  |  |  |  |
| Hong Chen et al 2005 | 3.14 | 2.67 | 7 | 8.22 | 2.99 | 9 |
| Minmin Zheng et al 2017 | 16.84 | 1.53 | 16 | 19.63 | 1.95 | 16 |
| Shen Shen et al 2018 | 22.43 | 1.23 | 10 | 36.94 | 1.52 | 10 |
| Zhongqiu Luan 2012 | 15.89 | 1.89 | 16 | 19.63 | 1.95 | 16 |
| Mice |  |  |  |  |  |  |
| Hong Chen et al 2021 | 30.75 | 6.54 | 10 | 50.79 | 5.74 | 10 |
| Huihui Wang et al 2019 | 2.75 | 0.15 | 10 | 8.05 | 1.26 | 10 |
| Qiannan Song et al 2022 | 1.295 | 0.042 | 20 | 1.521 | 0.091 | 20 |
| Siyan Ran 2017 | 0.421 | 0.085 | 15 | 0.723 | 0.088 | 15 |

**S-Table2. Data of TGF-β1 expression extracted from included studies between treatment group and blank control group.**

| Study or Subgroup | Experimental | | total | Control | | total |
| --- | --- | --- | --- | --- | --- | --- |
|  | mean | SD |  | mean | SD |  |
| Hong Chen et al 2005 | 3.14 | 2.67 | 7 | 1.26 | 6 | 6 |
| Minmin Zheng et al 2017 | 16.84 | 1.53 | 16 | 10.95 | 1.22 | 12 |
| Shen Shen et al 2018 | 22.43 | 1.23 | 10 | 11.9 | 1.05 | 10 |
| Zhongqiu Luan 2012 | 15.89 | 1.89 | 16 | 10.92 | 1.22 | 12 |
| Hong Chen et al 2021 | 30.75 | 6.54 | 10 | 18.92 | 3.43 | 10 |
| Huihui Wang et al 2019 | 2.75 | 0.15 | 10 | 1.75 | 0.05 | 10 |
| Qiannan Song et al 2022 | 1.295 | 0.042 | 20 | 1 | 0.091 | 20 |
| Siyan Ran 2017 | 0.421 | 0.085 | 15 | 0.239 | 0.054 | 15 |

**S-Table3. Data of α-SMA expression extracted from included studies between treatment group and animal model control group.**

| Study or Subgroup | Experimental | | total | Control | | total |
| --- | --- | --- | --- | --- | --- | --- |
|  | mean | SD |  | mean | SD |  |
| Rats |  |  |  |  |  |  |
| Hong Chen et al 2005 | 3.43 | 3.95 | 7 | 8.01 | 3.28 | 9 |
| Shen Shen et al 2018 | 32.5 | 1.02 | 10 | 35.72 | 1.2 | 10 |
| Zhongqiu Luan 2012 | 16.2 | 1.86 | 16 | 20.61 | 2.23 | 10 |
| Mice |  |  |  |  |  |  |
| Huihui Wang et al 2019 | 1.78 | 0.041 | 10 | 7.97 | 2.12 | 10 |
| Qiannan Song et al 2022 | 1.463 | 0.047 | 20 | 1.739 | 0.101 | 20 |

**S-Table4. Data of α-SMA expression extracted from included studies between treatment group and blank control group.**

| Study or Subgroup | Experimental | | total | Control | | total |
| --- | --- | --- | --- | --- | --- | --- |
|  | mean | SD |  | mean | SD |  |
| Hong Chen et al 2005 | 3.43 | 3.95 | 7 | 1.5 | 0.84 | 6 |
| Shen Shen et al 2018 | 32.5 | 1.02 | 10 | 23.89 | 1.75 | 10 |
| Zhongqiu Luan 2012 | 16.2 | 1.86 | 16 | 14.46 | 2.42 | 12 |
| Huihui Wang et al 2019 | 1.78 | 0.041 | 10 | 1.05 | 0.05 | 10 |
| Qiannan Song et al 2022 | 1.463 | 0.047 | 20 | 1 | 0.019 | 20 |

**S-Table5. Data of Scr expression level extracted from included studies between treatment group and animal model control group.**

| Study or Subgroup | Experimental | | total | Control | | total |
| --- | --- | --- | --- | --- | --- | --- |
|  | mean | SD |  | mean | SD |  |
| Rats |  |  |  |  |  |  |
| Minmin Zheng et al 2015 | 66.73 | 5.89 | 6 | 85.67 | 8.15 | 6 |
| Zhongqiu Luan 2012 | 66.73 | 5.89 | 16 | 85.67 | 8.15 | 16 |
| Mice |  |  |  |  |  |  |
| Shen Shen et al 2018 | 70.07 | 8.9 | 10 | 73.95 | 9.69 | 10 |
| Siyan Ran 2017 | 26.63 | 14.34 | 15 | 33.3 | 11.31 | 15 |

**S-Table6. Data of Scr expression level extracted from included studies between treatment group and blank control group.**

| Study or Subgroup | Experimental | | total | Control | | total |
| --- | --- | --- | --- | --- | --- | --- |
|  | mean | SD |  | mean | SD |  |
| Shen Shen et al 2018 | 70.07 | 8.9 | 10 | 71.78 | 10.74 | 10 |
| Zhongqiu Luan 2012 | 66.73 | 5.89 | 16 | 35.76 | 4.58 | 12 |
| Minmin Zheng et al 2015 | 66.73 | 5.89 | 6 | 35.76 | 4.58 | 6 |
| Siyan Ran 2017 | 26.63 | 14.34 | 15 | 21.38 | 14.94 | 15 |

**S-Table7. Data of BUN expression level extracted from included studies between treatment group and animal model control group.**

| Study or Subgroup | Experimental | | total | Control | | total |
| --- | --- | --- | --- | --- | --- | --- |
|  | mean | SD |  | mean | SD |  |
| Shen Shen et al 2018 | 6.08 | 1.44 | 10 | 8.16 | 0.41 | 10 |
| Zhongqiu Luan 2012 | 13.62 | 0.98 | 16 | 15.37 | 1.53 | 16 |
| Siyan Ran 2017 | 7.71 | 1.27 | 15 | 10.22 | 2.62 | 15 |
| Minmin Zheng et al 2015 | 13.62 | 0.98 | 6 | 15.37 | 1.53 | 6 |

**S-Table8. Data of BUN expression level extracted from included studies between treatment group and blank control group.**

| Study or Subgroup | Experimental | | total | Control | | total |
| --- | --- | --- | --- | --- | --- | --- |
|  | mean | SD |  | mean | SD |  |
| Shen Shen et al 2018 | 6.08 | 1.44 | 10 | 6.16 | 0.82 | 10 |
| Zhongqiu Luan 2012 | 13.62 | 0.98 | 16 | 9.16 | 0.78 | 12 |
| Siyan Ran 2017 | 7.71 | 1.27 | 15 | 7.41 | 2.4 | 15 |
| Minmin Zheng et al 2015 | 13.62 | 0.98 | 6 | 9.16 | 0.78 | 6 |

**S-Table9. Data of tissue inflammation score extracted from included studies between treatment group and animal model control group.**

| Study or Subgroup | Experimental | | total | Control | | total |
| --- | --- | --- | --- | --- | --- | --- |
|  | mean | SD |  | mean | SD |  |
| Hong Chen et al 2021 | 1.32 | 0.17 | 10 | 1.92 | 0.39 | 10 |
| Jingjin Yang 2016 | 1.33 | 0.52 | 6 | 3.17 | 1.17 | 6 |
| Li Sheng et al 2006 | 8.66 | 1.2 | 5 | 17.09 | 5.58 | 5 |
| Qiufeng Tang 2014 | 23.12 | 8.1 | 8 | 38.24 | 17.21 | 8 |
| Ting Yu 2018 | 5.53 | 0.08 | 15 | 7.01 | 0.33 | 15 |

**S-Table10. Data of tissue inflammation score extracted from included studies between treatment group and blank control group.**

| Study or Subgroup | Experimental | | total | Control | | total |
| --- | --- | --- | --- | --- | --- | --- |
|  | mean | SD |  | mean | SD |  |
| Hong Chen et al 2021 | 1.32 | 0.17 | 10 | 0.52 | 0.12 | 10 |
| Jingjin Yang 2016 | 1.33 | 0.52 | 6 | 0.33 | 0.52 | 6 |
| Li Sheng et al 2006 | 8.66 | 1.2 | 5 | 7.38 | 0.69 | 5 |
| Qiufeng Tang 2014 | 23.12 | 8.1 | 8 | 5.59 | 0.88 | 8 |
| Ting Yu 2018 | 5.53 | 0.08 | 15 | 4.56 | 0.1 | 15 |
